# Supplementary material for: Discovery of an Orally Effective Factor IX-Transferrin Fusion Protein for Hemophilia B
Source: Int J Mol Sci. 2019 Dec 18;21(1):21. doi: 10.3390/ijms21010021 (PMC6981973; doi:10.3390/ijms21010021)
Supplement: Supplementary file 1 [file ijms-21-00021-s001.zip › Figure S1_supporting information.pdf]

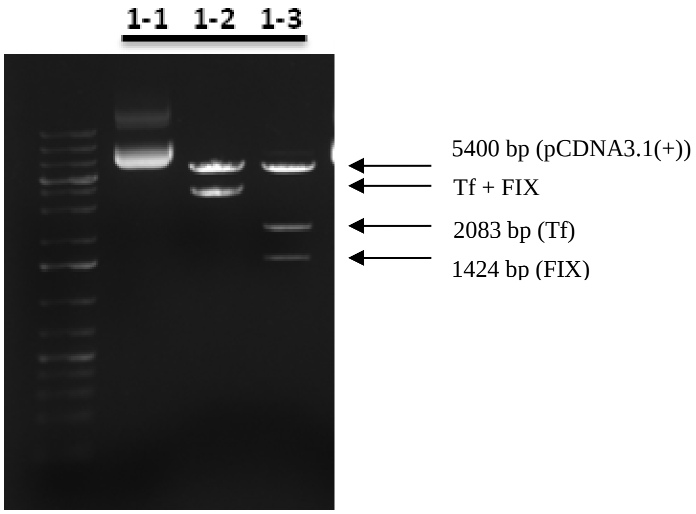

1-1: FIX-Tf-pCDNA3.1(+)
1-2: FIX-Tf-pCDNA3.1(+) AFLII and XbaI digestion
1-3: FIX-Tf-pCDNA3.1(+) AFLII, XhoI and XbaI digestion

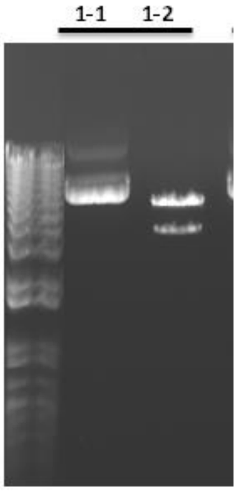

1-1: FIX-A(EAAAA)5A-Tf-pCDNA3.1(+)
1-2: FIX-A(EAAAA)5A-Tf-pCDNA3.1(+) AFLII and XbaI digestion

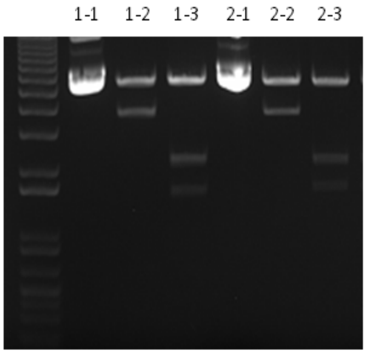

1-1. FIX-A(EAAAA)2A-Tf-pCDNA3.1(+)
1-2.FIX-A(EAAAA)2A-Tf-pCDNA3.1(+) digested with AflII and XbaI
1-3.FIX-A(EAAAA)2A-Tf-pCDNA3.1(+) digested with AflII ,xhoI and XbaI
2-1.FIX-SVSQTSKLTRAETVFPDVDGS-Tf-pCDNA3.1(+)
2-2.FIX-SVSQTSKLTRAETVFPDVDGS-Tf-pCDNA3.1(+) digested with AfLII and XbaI
2-3.FIX-SVSQTSKLTRAETVFPDVDGS-Tf-pCDNA3.1(+) _2 digested with AflII ,xhoI and XbaI

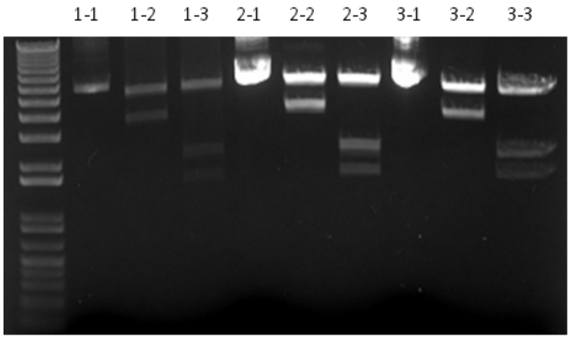

1-1. FIX-(GGGGS)2-Tf-pCDNA3.1(+)
1-2.FIX-(GGGGS)2-Tf-pCDNA3.1(+) digested with AflII and XbaI
1-3.FIX-(GGGGS)2-Tf-pCDNA3.1(+) digested with AflII ,xhoI and XbaI
2-1.FIX-dithiocyclopeptide-Tf-pCDNA3.1(+)
2-2.FIX-dithiocyclopeptide-Tf-pCDNA3.1(+) digested with AfLII and XbaI
2-3.FIX-dithiocyclopeptide-Tf-pCDNA3.1(+) digested with AflII ,xhoI and XbaI
3-1.FIX-(GGGGS)5-Tf-pCDNA3.1(+)
3-2.FIX-(GGGGS)5-Tf-pCDNA3.1(+) digested with AfLII and XbaI
3-3.FIX-(GGGGS)5-Tf-pCDNA3.1(+)(+) digested with AflII ,xhoI and XbaI

Supporting Fig 1. Identification of FIX-Tf-pCDNA3.1(+) and various FIX-Linker-Tf-pCDNA 3.1(+) expression constructs by digestion with endonucleases.
