# Supplementary material for: Discovery of an Orally Effective Factor IX-Transferrin Fusion Protein for Hemophilia B
Source: Int J Mol Sci. 2019 Dec 18;21(1):21. doi: 10.3390/ijms21010021 (PMC6981973; doi:10.3390/ijms21010021)
Supplement: Supplementary file 1 [file ijms-21-00021-s001.zip › Figure S2_supporting information.pdf]

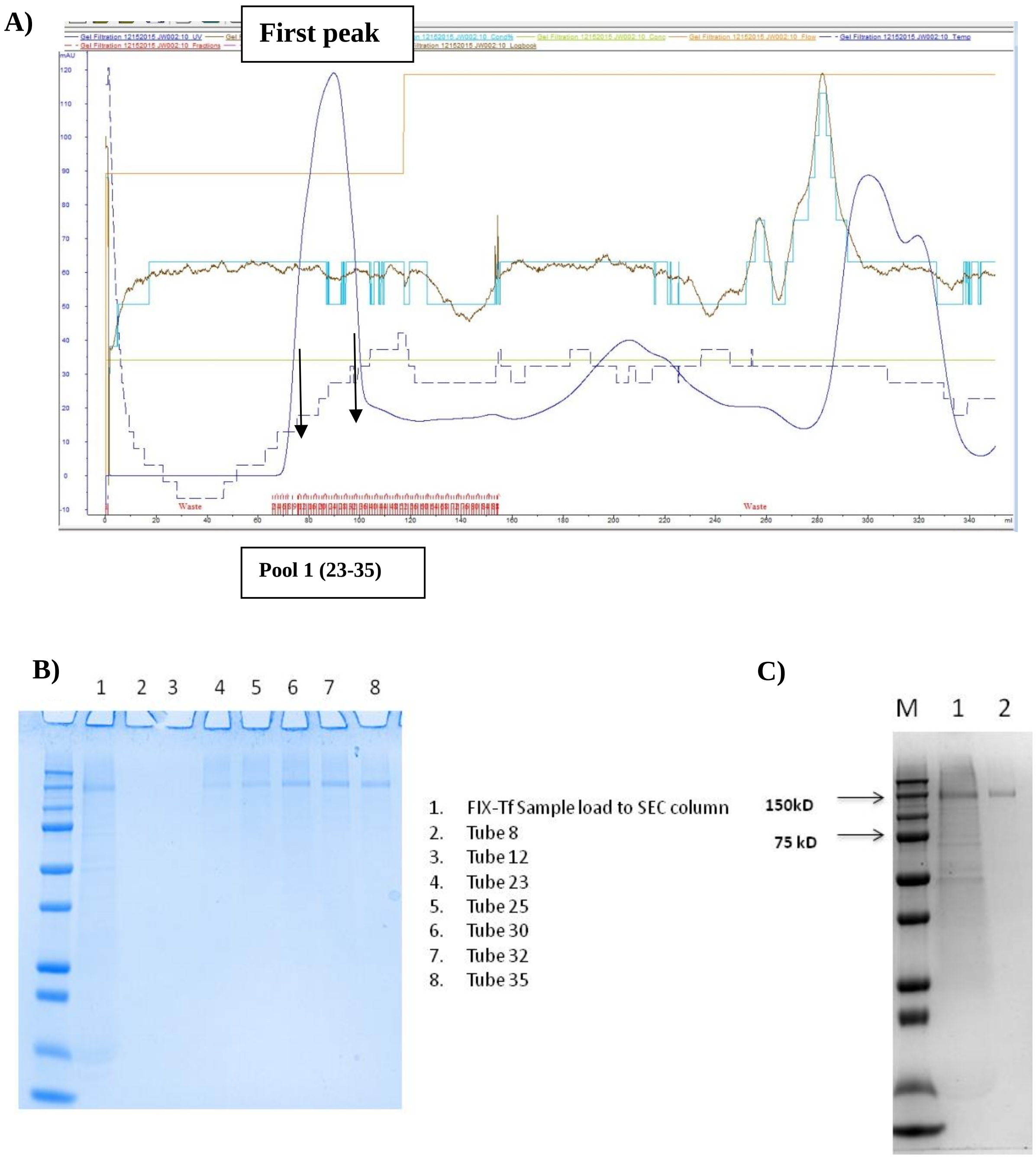

Supporting Fig 2. Separation of FIX-Tf fusion protein by size exclusion chromatography (SEC) (a) The protein eluted in the first peak (fraction 23-35) was pooled. (b) SDS-PAGE of protein samples in different SEC collection tubes. (c) SDS-PAGE of protein samples before and after purification by SEC. Marker proteins and their corresponding molecular masses are indicated. Lane 1, protein loading sample; lane 2, concentrated protein sample after purification by SEC.
